# A scoping review of Australian allied health research in ehealth

**DOI:** 10.1186/s12913-016-1791-x

**Published:** 2016-10-04

**Authors:** Teresa Iacono, Kellie Stagg, Natalie Pearce, Alana Hulme Chambers

**Affiliations:** 1 La Trobe Rural Health School, La Trobe University, PO Box 199, Bendigo, VIC 3550 Australia; 2 La Trobe University, PO Box 199, Bendigo, VIC 3550 Australia; 3 Department of Rural Health, University of Melbourne, Docker Street, Wangaratta, VIC 3677 Australia

**Keywords:** Ehealth, Telehealth, Telecare, Telepractice, Telerehabilitation, Allied health, Speech pathology, Occupational therapy, Podiatry, Physiotherapy, Social work, Dietetics, Exercise physiology

## Abstract

**Background:**

Uptake of e-health, the use of information communication technologies (ICT) for health service delivery, in allied health appears to be lagging behind other health care areas, despite offering the potential to address problems with service access by rural and remote Australians. The aim of the study was to conduct a scoping review of studies into the application of or attitudes towards ehealth amongst allied health professionals conducted in Australia.

**Methods:**

Studies meeting inclusion criteria published from January 2004 to June 2015 were reviewed. Professions included were audiology, dietetics, exercise physiology, occupational therapy, physiotherapy, podiatry, social work, and speech pathology. Terms for these professions and forms of ehealth were combined in databases of CINAHL (EBSCO), Cochrane Library, PsycINFO (1806 – Ovid), MEDLINE (Ovid) and AMED (Ovid).

**Results:**

Forty-four studies meeting inclusion criteria were summarised. They were either trials of aspects of ehealth service delivery, or clinician and/or client use of and attitudes towards ehealth. Trials of ehealth were largely from two research groups located at the Universities of Sydney and Queensland; most involved speech pathology and physiotherapy. Assessments through ehealth and intervention outcomes through ehealth were comparable with face-to-face delivery. Clinicians used ICT mostly for managing their work and for professional development, but were reticent about its use in service delivery, which contrasted with the more positive attitudes and experiences of clients.

**Conclusion:**

The potential of ehealth to address allied health needs of Australians living in rural and remote Australia appears unrealised. Clinicians may need to embrace ehealth as a means to radicalise practice, rather than replicate existing practices through a different mode of delivery.

**Electronic supplementary material:**

The online version of this article (doi:10.1186/s12913-016-1791-x) contains supplementary material, which is available to authorized users.

## Background

*ehealth*, the use of information communication technologies (ICT) for health service delivery [1], offers the potential to improve efficiencies and quality in health care delivery in the face of increasing costs and skilled health workforce shortages. Systematic reviews have demonstrated the successful use of ehealth, such as in providing services to aged care residents [2], and for remote stroke assessment and rehabilitation [3]. Arguably, people in rural and remote Australia, in particular, stand to benefit from ehealth [4], with outcomes at least equivalent to face-face care [2, 5] and the potential to enhance continuity of care [5]. Despite a burgeoning of ehealth applications, the focus of reviews has been medical and psychiatric applications [4], with little in allied health. Edirippulige et al. [2] for example, identified 5 of 22 studies involving ehealth in allied health services for residents in aged care.

In Australia, there are strong arguments for ehealth in allied health. Limited service provision to rural and remote Australia has been associated with poor recruitment and retention of allied health clinicians [6–8]. Tertiary education initiatives, such as drawing students from rural areas, have seen an increase in graduates taking up rural positions, but failed to solve the problem [9, 10]. Veitch et al. [10] suggested that technology offers one solution, but findings from a national survey of 1125 practitioners and 40 interviews from 15 allied health professions, while indicating support for ehealth, also indicated limited scope and frequency of use [11]. Respondents fell along a continuum from strong supporters to non-adopters. Even those making ready use of ehealth did so mainly for practice administration and professional research and development, with much less consideration given to patient or consumer benefits or perspectives. It has been suggested that such limited use may be linked to a lack of relevant ehealth content in undergraduate allied health curriculum [12].

A high level of access to the internet amongst Australians (83/100) [13] demonstrates a vector through which the benefits of ehealth for rural and remote areas can be realised, particularly assisted by the National Broadband Network (NBN). Unfortunately, the specific mechanisms and nature of the infrastructure have been disputed across governments, resulting in delays in the roll out and inequalities in access [14]. Certainly, until the NBN reaches all parts of Australia, people living in rural and regional areas will continue to experience discrepancies with those living in urban locations in terms of reliance on mobile wireless technology (with some areas being susceptible to dead spots), satellite broadband, and even in some cases, telephone dial-up [15]. A review of empirical evidence to support ehealth uptake can provide a basis for innovation in allied health provision in the Australian context of inequalities in both health and internet access. There is a need to address clinician concerns about whether benefits, in terms of professional collaboration, continuity of care and practice efficiency, outweigh costs and perceived barriers, including risks to patient privacy, productivity and patient-practitioner relationships [11].

### Aims

We conducted a scoping review of the empirical support for allied ehealth through Australian applications. The specific questions were: (1) What aspects of ehealth use by allied health clinicians have been addressed in research conducted in Australia? and (2) What are the outcomes of this research?

## Methods

### Ethical approval

This review did not involve primary research involving humans, hence approval from an ethics committee was not sought.

### Design

Scoping reviews address the purpose of this study: that is, to examine the nature and extent of research about a topic or to answer a question [16, 17]. We followed the framework of Arskey, OMalley [16], involving conducting a systematic search from which studies relevant to the questions posed are selected, charting the data according to key issues or themes, and summarizing and reporting the data.

### Search and study selection

We adopted the World Health Organization definition of ehealth as the use of ICT for the delivery of health services [1]. A selection of allied health professions that provided variation in care practice [11] were included: audiology, dietetics, exercise physiology, occupational therapy, physiotherapy, podiatry, social work, and speech pathology. Terms for these professions and forms of ehealth were combined: telemedicine, telerehabilitation, telehealth, telecare, M-health, E-health, ICT, health; combined with allied health, speech therap*, speech patholog*, occupational therap*, physiotherap*, physical therap*, podiatr*, exercise physiolog*, dietetic*, social work*, audiolog*. Studies from the previous 10 years captured the recent expansion of internet applications in healthcare [18]. Searched databases were CINAHL (EBSCO), Cochrane Library, PsycINFO (1806 – Ovid), MEDLINE (Ovid) and AMED (Ovid).

**Fig. 1:**
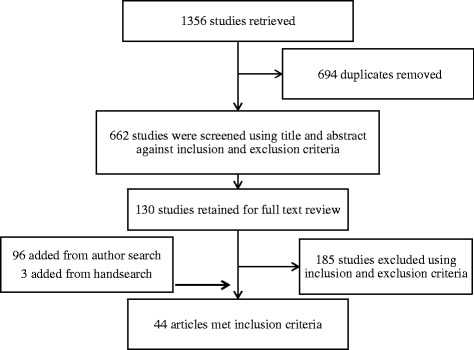
Search selection process and outcomes

Included were primary studies conducted in any part of Australia, involving one or more allied health profession that directly addressed any aspect of and/or attitude towards ehealth, published from January 2004 to June 2015. Excluded were conference proceedings, books/book sections, and journal articles without primary data, those whose primary focus was not ehealth, or data specific to allied health practitioners was not provided. The electronic search was supplemented by hand searches of included studies and the journals *Telehealth and Telemedicine*, and *Telemedicine* and *e-health*. Figure 1 shows the search and selection process that yielded 44 studies.

### Data extraction

Each study was summarised according to citation, participant group and clinical problem, study focus and design, ICT employed, and key outcomes. Authors TI, KS and AHC completed the study extraction; to check for consistency, TI and KS both summarised three of the studies, and TI and AHC both summarised two of the studies. Disagreements about studies meeting inclusion criteria were resolved through consensus discussion by TI, KS and AHC.

## Results

**Fig. 2:**
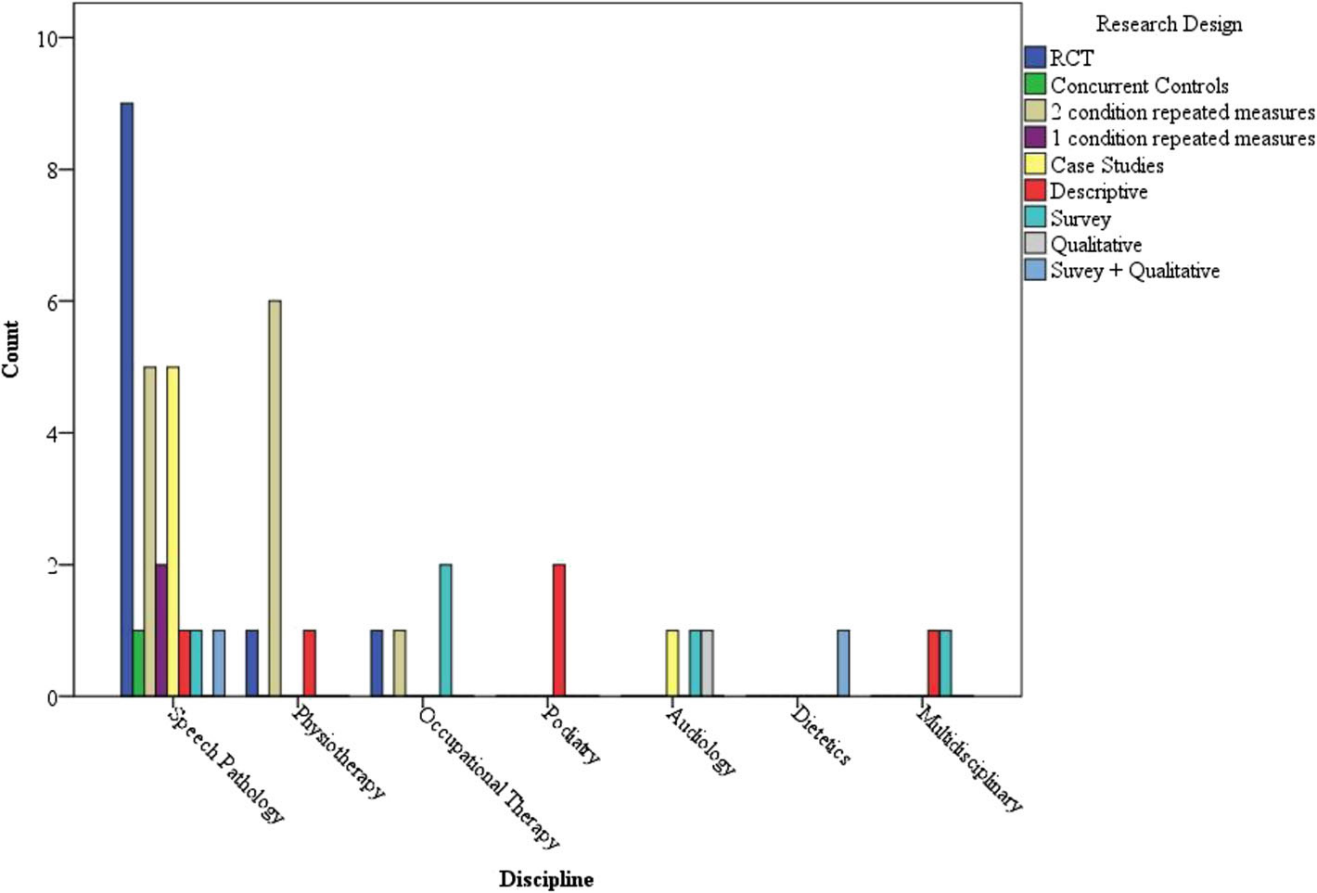
Allied health disciplines represented in studies according to research designs employed

Studies were broadly categorised as either direct trials of ehealth (*n* = 33), or surveys and qualitative studies providing clinician and in some cases client opinions (*n* = 11). Study designs for the direct trials were descriptive outcome studies of groups (*n* = 5) or cases (*n* = 6), single group comparisons across conditions (*n* = 10), comparison across treatment and control groups (without randomisation, *n* = 1), or Randomised Controlled Trials (RCT, *n* = 11). Figure 2 provides an illustration of the disciplines represented across studies according to study type. (Summaries of studies can be found in Additional file 1.)

### Trials

Inspection of Fig. 2 shows that most direct trials were of speech pathology applications (64 %), followed by physiotherapy (22 %), with only one study in each of occupational therapy, podiatry and audiology. In one study, both speech pathologists and physiotherapists participated as part of telerehabilitation teams. No direct ehealth applications were identified for the other allied health professions.

The speech pathology studies were largely from two research groups from The University of Sydney (USyd) and The University of Queensland (UQ). The USyd focus was on telehealth for evidence-based stuttering interventions for children [19–21], adolescents [22, 23], and adults [24, 25]. They reflected technology developments from telephone to the internet, beginning with phase 1 trials to demonstrate viability [19, 21, 22, 24], then progressing to RCT [21, 26]. Telehealth was found to be at least as effective as therapy delivered face-to-face, with both parent and client satisfaction reported [19–23].

The UQ studies addressed speech pathology assessment and treatment of a variety of disorders across child [26–28] and adult client groups using telerehabilitation: a computer with videoconferencing capability, and software designed for purpose, allowing real-time data capture, store and forward. Remote communication was simulated in most studies using dial-up or wireless internet to deliver low bandwidth videoconferencing to connect clinicians and clients located in the same building, argued to reflect the technology most available to rural and remote communities [37, 41]. Many studies were RCT, and demonstrated good agreement between measures recorded or clinician judgements made across face-to-face and the telerehabilitation system, and one study demonstrated non-inferiority of telerehabilitation compared to face-to-face delivery [30]. Also reported were client satisfaction and willingness to experience this form of service delivery again [30, 36, 39, 42]. Ward et al. [36] found that clinician satisfaction was not as high as that obtained for clients who received communication and swallowing assessment post-laryngectomy.

Six of the eight studies in physiotherapy [41, 43–47] and one in occupational therapy [48] were also from the UQ group. Similar patterns were evident to the speech pathology applications: (a) varied problems (e.g., knee, shoulder, elbow problems) across client groups (e.g., Parkinson’s Disease, Cystic Fibrosis); and (b) use of similar telerehabilitation components, again with low band-width telephone dial-up or wireless technology connecting parties within the same building. Most studies focused on agreement across delivery modes in terms of diagnostic assessments and intra- and inter-reliability for on-line ratings or evaluations [43–46], with another two studies in physiotherapy conducted at La Trobe University [49, 50], one of which demonstrated a similar focus and findings [49]. Designs employed were mostly repeated measures across remote and face-to-face conditions [43–46, 49], with descriptive outcome statistics provided in one study [50]. Of the two intervention studies, Russell et al. [41] found no differences in outcomes and compliance across face-to-face and telerehabilitation delivery for clients following total knee arthroplasty using an RCT. Further, clients receiving telerehabilitation reported high levels of satisfaction. Holland et al. [50] reported significant improvements for five of eight patients with chronic obstructive pulmonary disease following telerehabilitation, without adverse events across all patients.

In the one podiatry study [51], videoconferences and emails connected clinicians and clients located in a remote part of Western Australia and an expert clinician at a metropolitan hospital. They used hi fidelity digital imagery to share information about the healing of diabetic foot ulcers. Positive client outcomes and lack of resistance by local clinicians were reported. Similarly, Pearce et al. [52] demonstrated the feasibility of telehealth delivery of audiology services to patients located in rural and remote locations.

In the only direct trial study involving more than one allied health discipline, Crotty and colleagues [53] evaluated the feasibility of telerehabilitation, provided largely by speech pathologists and physiotherapists, for community rehabilitation and rural nursing home patients. Descriptive data indicated good outcomes for most patients in terms of achieving rehabilitation goals, and acceptance by clients as well as clinicians, who also benefited from reduced time in travel. This study was notable for the use of off-the-shelf technology, such as tablet devices, rather than dedicated telerehabilitation equipment as used in many studies.

### Attitudes and opinions

Most surveys and qualitiative studies focused on attitudes towards and use of ehealth by speech pathologists [54] and occupational therapists [55–57] in the absence of implementation of ehealth with clients. Therapists did not tend to engage in ehealth, rather using ICT for work management tasks and professional development. Barriers included lack of access to and support in ICT. Concerns were raised about poor client-clinician relationships resulting from teleconference-delivery [55], but clinician respondents to Dunkley et al. [54] perceived clients living in rural areas to be more negative about the use of ICT than was reported by clients. The lack of ehealth use and negative attitudes towards it occurred even though some study participants delivered services across large geographic areas [55, 56]. However, results by Taylor, Lee [57] suggested that rural speech pathologists were more likely to use ehealth than were their metropolitan colleagues. One survey study addressed client attitudes towards the delivery of audiology services by telehealth [58], which indicated a general lack of awareness of this form of service delivery and a preference for face-to-face consultations. Further, in a study involving a podiatry clinic providing chronic wound care in rural Western Australia, barriers to ehealth included delays in installing software and losing staff trained in the technologies because of staff turn-over [59].

Two studies addressed specific aspects of ehealth following its implementation [60, 61]. Meyer et al. [60] found limited follow-up with professionals by adults who had failed a phone-delivered hearing screening. In one of few studies to explore the ehealth experiences of clients, Constantinescu [61] found that parents of children with hearing impairments were satisfied with speech pathology intervention delivered by teleconference, and reported good child-therapist interaction, despite some technical difficulties affecting audio quality. The clinicians were similarly comfortable with the therapy sessions, but had fewer technical difficulties.

The only study involving dieticians related to use of an ehealth records prototype to assist in the adoption of international terminology standards [62]. Fewer than half the participants (*n* = 5) trialled the prototype, resulting in only a small increase in confidence in using the terminology. Finally, in one study including a number of allied health professions, attitudes towards website use to provide information to clients was explored [63]. There was limited use by professionals, with social workers and dieticians most likely to provide clients with information from websites.

## Discussion

Research into Australian ehealth in allied health has been limited in scope. Efficacy studies, conducted mostly in speech pathology and physiotherapy, have demonstrated reliable and valid assessment, and similar intervention outcomes across ehealth and face-to-face delivery, but the actual experience of ehealth was at best a simulated one in many studies. A picture that emerges from this review and the large national survey [11] is of allied health practitioners being willing to use ICT to improve their work efficiency and for professional development, but reticent about using it for service delivery. This finding is at odds with client perspectives, which reflected a more positive attitude and willingness to participate in ehealth, although the study of audiology patients suggested a lack of awareness of tele-enabled service delivery and a preference for in-person consultations [58].

The focus on the fidelity of intervention and limited real-life ehealth applications with few attempts to understand the client perspective also fails to align with patient- or client-centred care. Further, evidence of limited use of the web to provide clients with information ignores a push towards self-managed care, facilitated by an increase in health service users’ reliance on the internet for health-related information [63].

Limited uptake of ehealth may be attributable to a desire for new technologies to support existing ways of working, rather than embracing its potential to revolutionalise work practices [64]. Without a strong grounding in ehealth, clinicians may lack the skills and insights needed [12], or to prepare them to radically change their ways of working [11]. Rather, clinicians appear to rely on ICT to increase the efficiency of their current practices, with those willing to embrace it for other purposes being thwarted by a lack of ICT support, whether real or perceived [11]. Additional factors influencing clinician adoption of ICT may include their access to the required equipment within the work setting, as well as system-level supports (commitment of managers, investment of the organisation) as much as attitude of the practitioner.

### Limitations and directions for further research

We addressed only a subset of allied health professions. Still, the clinician uses and attitudes in these studies reflect the findings of the large survey with a more comprehensive list of professions [11]. As with all systematic searches, it is also possible that our terms failed to capture all studies that would have met inclusion criteria. Certainly, many terms have been used to reflect all possible uses of ICT for health service delivery. Still, our combination of data-based and hand searches suggest that the final selection did enable a true reflection of the scope of research in this area within the Australian context.

The concerns clinicians have about ehealth [11] have not been directly addressed, at least in the Australian context. Further research is needed into information privacy, how ehealth supports continuity of care and promotion of client-clinician relationships. There is also a need to involve ICT policy makers and professionals, and others involved in ehealth implementation to determine the veracity of clinician concerns about lack of support and resources [11, 55] and harness the evolution of ICT to facilitate ehealth delivery to meet varied needs. There is some evidence of early adopters of ehealth amongst allied health professionals [11], who perhaps can lead its uptake amongst colleagues who may be more reticent or require peer support. Advantages, such as saving time spent travelling to clients [53] can provide motivation for extending the benefits of ehealth, particularly for rural clients, providing the potential for more frequent contact and support from clinicians. Researching such initiatives would seem a logical next step, as well as into the client experience of ehealth in the context of variable speed of internet access across rural and remote Australia.

Finally, our review focused on the Australian context, which functions with a particular health care system, with ehealth reliant on internet access that varies in quality and reliability across parts of the country [14, 15]. International comparisons would shed light on the extent to which countries with health system differences make use of ICT to deliver allied health services to people with various access to, and perhaps level of acceptance of ehealth.

## Conclusions

The potential benefits of ehealth remain unrealised in allied health. Research has demonstrated that traditional allied health assessment and intervention services can be reliably and validly implemented through ehealth. This research provides a foundation on which to build ehealth initiatives, but does not address key concerns of clinicians or demonstrate innovative service delivery. Enlisting stakeholder support from communities who stand to benefit from ehealth and applying comprehensive research strategies may assist in shifting the research to exploring revolutionary practices that will deliver benefits to both service providers and recipients.

## Additional file

Additional file 1: Table S1. Summaries of studies with a focus on ehealth trials. In this table, the following information extracted from each study included in the scoping review has been presented: study citation, participant group/problem, design, technology used and key outcomes. Studies have been presented according allied health discipline groups [19–23, 25–53]. **Table S2**. Summaries of studies primarily of attitudes and opinions about ehealth. In this table, the following information extracted from each study included in the scoping review has been presented: study citation, participant group, focus, design, technology employed, and key outcomes. Studies have been presented according allied health discipline groups [54–63]. (DOCX 47 kb)

## Abbreviations

ICT: Information communication technologies
NBN: National broadband network
RCT: Randomised controlled trials
UQ: The University of Queensland
USyd: The University of Sydney

## References

[1] World Health Organization. ehealth. 2013. http://www.who.int/topics/ehealth/en/. Accessed 5 Feb 2014.

[2] Edirippulige S, Martin-Khan M, Beattie E, Smith AC, Gray LC (2013). A systematic review of telemedicine services for residents in long term care facilities. J Telemed Telecare.

[3] Johansson T, Wild C (2011). Telerehabilitation in stroke care - A systematic review. J Telemed Telecare.

[4] Banbury A, Roots A, Nancarrow S (2014). Rapid review of applications of e‐health and remote monitoring for rural residents. Aust J Rural Health.

[5] Kairy D, Lehoux P, Vincent C, Visintin M (2009). A systematic review of clinical outcomes, clinical process, healthcare utilization and costs associated with telerehabilitation. Disabil Rehabil.

[6] Campbell N, McAllister L, Eley D (2012). The influence of motivation in recruitment and retention of rural and remote allied health professionals: A literature review. Rural Remote Health.

[7] Chisholm M, Russell D, Humphreys J (2011). Measuring rural allied health workforce turnover and retention: What are the patterns, determinants and costs?. Aust J Rural Health.

[8] Williams E, D’Amore W, McMeeken J (2007). Physiotherapy in rural and regional Australia. Aust J Rural Health.

[9] Dew A, Bulkeley K, Veitch C, Bundy A, Gallego G, Lincoln M (2013). Addressing the barriers to accessing therapy services in rural and remote areas. Disabil Rehabil.

[10] Veitch C, Dew A, Bulkeley K, Lincoln M, Bundy A, Gallego G (2012). Issues affecting therapist workforce and serivce delivery in the disability sector in rural and remote New South Wales, Australia: perspectives of policy-makers, managers and senior therapists. Rural Remote Health.

[11] Department of Health and Ageing. The eHealth Readiness of Australia’s Allied Health Sector. 2011. http://www.health.gov.au/internet/publications/publishing.nsf/Content/ehealth-readiness-allied-toc. Accessed 26 Nov 2014.

[12] Gray K, Dattakumar A, Maeder A, Butler‐Henderson K, Chenery H. Advancing Ehealth education for the clinical health professions. Final Report 2014. 2014. http://olt.gov.au/project-coordinated-interprofessional-curriculum-renewal-ehealth-capability-clinical-health-professi. Accessed 20 Dec 2014.

[13] World Bank. The World Bank Data. n.d. http://data.worldbank.org/indicator/IT.NET.USER.P2/countries/1W?display=default. Accessed 5 Dec 2014.

[14] Wilken R, Nansen B, Kennedy J, Gibbs M, Arnold M. NBN benefits regional centres, but rural Australia is still left wanting. In: The Conversation. 2014. http://theconversation.com/nbn-benefits-regional-centres-but-rural-australia-is-still-left-wanting-34532. Accessed 26 Nov 2014.

[15] Regional Technologies Independent Review Committee, Government A (2015). Regional telecommunications review: Unlocking the potential in regional Australia.

[16] Arksey H, O’Malley L (2005). Scoping studies: towards a methodological framework. Int J Soc Res Methodol.

[17] Grant MJ, Booth A (2009). A typology of reviews: an analysis of 14 review types and associated methodologies. Health Inf Libr J.

[18] Rozenblum R, Bates D (2013). Patient-centred healthcare, social media and the internet: The perfect storm?. Qual Saf Health Care.

[19] Wilson L, Onslow M, Lincoln M (2004). Telehealth adaptation of the Lidcombe Program of Early Stuttering Intervention: five case studies. Am J Speech Lang Pathol.

[20] Lewis C, Packman A, Onslow M, Simpson J, Jones M (2008). A phase II trial of telehealth delivery of the Lidcombe Program of Early Stuttering Intervention. Am J Speech Lang Pathol.

[21] O’Brian S, Smith K, Onslow M (2014). Webcam delivery of the Lidcombe Program for early stuttering: A Phase I clinical trial. J Speech Lang Hear Res.

[22] Carey B, O’Brian S, Onslow M, Packman A, Menzies R (2012). Webcam delivery of the Camperdown Program for adolescents who stutter: A Phase I trial. Lang Speech Hear Serv Sch.

[23] Carey B, O’Brian S, Lowe R, Onslow M (2014). Webcam delivery of the Camperdown Program for adolescents who stutter: A Phase II trial. Lang Speech Hear Serv Sch.

[24] O’Brian S, Packman A, Onslow M (2008). Telehealth delivery of the camperdown program for adults who stutter: a phase I trial. J Speech Lang Hear Res.

[25] Carey B, O’Brian S, Onslow M, Block S, Jones M, Packman A (2010). Randomized controlled non-inferiority trial of a telehealth treatment for chronic stuttering: the Camperdown Program. Int J Lang Commun Disord.

[26] Waite M, Cahill L, Theodoros D, Busuttin S, Russell T (2006). A pilot study of online assessment of childhood speech disorders. J Telemed Telecare.

[27] Waite M, Theodoros D, Russell T, Cahill L (2010). Internet-based telehealth assessment of language using the CELF-4. Lang Speech Hear Serv Sch.

[28] Waite MC, Theodoros DG, Russell TG, Cahill LM (2012). Assessing children’s speech intelligibility and oral structures, and functions via an internet-based telehealth system. J Telemed Telecare.

[29] Constantinescu G, Theodoros D, Russell T, Ward E, Wilson S, Wootton R (2010). Assessing disordered speech and voice in Parkinson’s disease: a telerehabilitation application. Int J Lang Commun Disord.

[30] Constantinescu G, Theodoros D, Russell T, Ward E, Wilson S, Wootton R (2011). Treating disordered speech and voice in Parkinson’s disease online: a randomized controlled non-inferiority trial. Int J Lang Commun Disord.

[31] Hill A, Theodoros D, Russell T, Cahill L, Ward E, Clark K (2006). An internet-based telerehabilitation system for the assessment of motor speech disorders: a pilot study. Am J Speech Lang Pathol.

[32] Hill A, Theodoros D, Russell T, Ward E (2009). Using telerehabilitation to assess apraxia of speech in adults. Int J Lang Commun Disord.

[33] Sharma S, Ward E, Burns C, Theodoros D, Russell T (2011). Assessing swallowing disorders online: a pilot telerehabilitation study. Telemed J E Health.

[34] Theodoros D, Constantinescu G, Russell T, Ward E, Wilson S, Wootton R (2006). Treating the speech disorder in Parkinson’s disease online. J Telemed Telecare.

[35] Ward E, Crombie J, Trickey M, Hill A, Theodoros D, Russell T (2009). Assessment of communication and swallowing post-laryngectomy: A telerehabilitation trial. J Telemed Telecare.

[36] Ward L, White J, Russell T, Theodoros D, Kuhl M, Nelson K (2007). Assessment of communication and swallowing function post laryngectomy: A telerehabilitation trial. J Telemed Telecare.

[37] Ward EC, Sharma S, Burns C, Theodoros D, Russell T (2012). Validity of Conducting Clinical Dysphagia Assessments for Patients with Normal to Mild Cognitive Impairment via Telerehabilitation. Dysphagia.

[38] Constantinescu GA, Theodoros DG, Russell TG, Ward EC, Wilson SJ, Wootton R (2010). Home-based speech treatment for Parkinson’s disease delivered remotely: A case report. J Telemed Telecare.

[39] Theodoros D, Hill A, Russell T, Ward E, Wootton R (2008). Assessing acquired language disorders in adults via the Internet. Telemed J E Health.

[40] Hill AJ, Theodoros DG, Russell TG, Ward EC, Wootton R (2009). The effects of aphasia severity on the ability to assess language disorders via telerehabilitation. Aphasiology.

[41] Russell T, Buttrum P, Wootton R, Jull GA (2011). Internet-based outpatient telerehabilitation for patients following total knee arthroplasty: a randomized controlled trial. J Bone Joint Surg (Am Vol).

[42] Burns C, Ward E, Hill A, Malcolm K, Bassett L, Kenny L (2012). A pilot trial of a speech pathology telehealth service for head and neck cancer patients. J Telemed Telecare.

[43] Lade H, McKenzie S, Steele L, Russell T (2012). Validity and reliability of the assessment and diagnosis of musculoskeletal elbow disorders using telerehabilitation. J Telemed Telecare.

[44] Russell T, Blumke R, Richardson B, Truter P (2010). Telerehabilitation mediated physiotherapy assessment of ankle disorders. Physiother Res Int.

[45] Steele L, Lade H, McKenzie S, Russell TG. Assessment and diagnosis of musculoskeletal shoulder disorders over the internet. International Journal of Telemedicine and Applications. 2012. doi:10.1155/2012/945745 10.1155/2012/945745 PMC3501948 23193395

[46] Russell T, Hoffmann T, Nelson M, Thompson L, Vincent A (2013). Internet-based physical assessment of people with Parkinson disease is accurate and reliable: A pilot study. J Rehabil Res Dev.

[47] Russell T, Truter P, Blumke R, Richardson B (2010). The diagnostic accuracy of telerehabilitation for nonarticular lower-limb musculoskeletal disorders. Telemed J E Health.

[48] Hoffmann T, Russell T, Thompson L, Vincent A, Nelson M (2008). Using the Internet to assess activities of daily living and hand function in people with Parkinson’s disease. NeuroRehabilitation.

[49] Cox N, Alison J, Butto B, Wilson J, Holland A (2013). Assessing exercise capacity using telehealth: A feasibility study in adults with cystic fibrosis. Respir Care.

[50] Holland A, Hill C, Rochford P, Fiore J, Berlowitz D, McDonald C (2013). Telerehabilitation for people with chronic obstructive pulmonary disease: Feasibility of a simple, real time model of supervised exercise training. J Telemed Telecare.

[51] Manuel P (2012). A prospective, interventional study of the effectiveness of digital wound imaging, remote consultation and podiatry offloading devices on the healing rates of chronic lower extremity wounds in remote regions of Western Australia. Wound Pract Res.

[52] Pearce W, Ching TY, Dillon H (2009). A pilot investigation into the provision of hearing services using tele-audiology to remote areas. Aust N Z J Audiol.

[53] Crotty M, Killington M, van den Berg M, Morris C, Taylor A, Carati C (2014). Telerehabilitation for older people using off-the-shelf applications: acceptability and feasibility. J Telemed Telecare.

[54] Dunkley C, Pattie L, Wilson L, McAllister L (2010). A comparison of rural speech-language pathologists’ and residents’ access to and attitudes towards the use of technology for speech-language pathology service delivery. Int J Speech Lang Pathol.

[55] Chedid R, Dew A, Veitch C (2013). Barriers to the use of Information and Communication Technology by occupational therapists working in a rural area of New South Wales, Australia. Aust Occup Ther J.

[56] Hoffmann T, Cantoni N (2008). Occupational therapy services for adult neurological clients in Queensland and therapists’ use of telehealth to provide services. Aust Occup Ther J.

[57] Taylor R, Lee H (2005). Occupational therapists’ perception of usage of information and communication technology (ICT) in Western Australia and the association of availability of ICT on recruitment and retention of therapists working in rural areas. Aust Occup Ther J.

[58] Eikelboom RH, Atlas MD (2005). Attitude to telemedicine, and willingness to use it, in audiology patients. J Telemed Telecare.

[59] Barrett M, Larson A, Carville K, Ellis I (2009). Challenges faced in implementation of a telehealth enabled chronic wound care system. Rural Remote Health.

[60] Meyer C, Hickson L, Khan A, Hartley D, Dillon H, Seymour J (2011). Investigation of the actions taken by adults who failed a telephone-based hearing screen. Ear Hear.

[61] Constantinescu G (2012). Satisfaction with telemedicine for teaching listening and spoken language to children with hearing loss. J Telemed Telecare.

[62] O’Sullivan T (2013). Evaluation of an electronic record prototype incorporating the Nutrition Care Process and International Dietetics and Nutrition Terminology. Nutr Diet.

[63] Usher W (2011). Developing policies for e-health: use of online health information by Australian health professionals and their patients. HIM J.

[64] Westbrook J, Braithwaite J (2010). Will information and communication technology disrupt the health system and deliver on its promise?. Med J Aust.

