# Supplementary material for: A scoping review of Australian allied health research in ehealth
Source: BMC Health Serv Res. 2016 Oct 4;16:543. doi: 10.1186/s12913-016-1791-x (PMC5050606; doi:10.1186/s12913-016-1791-x)
Supplement: Additional file 1: Table S1. — Summaries of studies with a focus on ehealth trials. In this table, the following information extracted from each study included in the scoping review has been presented: study citation, participant group/problem, design, technology used and key outcomes. Studies have been presented according allied health discipline groups [19–23, 25–53]. Table S2. Summaries of studies primarily of attitudes and opinions about ehealth. In this table, the following information extracted from each study included in the scoping review has been presented: study citation, participant group, focus, design, technology employed, and key outcomes. Studies have been presented according allied health discipline groups [54–63]. (DOCX 47 kb) [file 12913_2016_1791_MOESM1_ESM.docx]

Table 1.

*Summaries of studies with a focus on ehealth trials*

| *Study* | *Participant Group/ Problem* | *Focus* | *Design* | *Technology* | *Key Outcomes* |
| --- | --- | --- | --- | --- | --- |
| *Speech Pathology* | | | | | |
| Wilson et al. [19] | Child stutterers (*n*=5) | Trial telehealth delivery of established early stuttering intervention | Case studies | Telephone  Audio recordings | - All participants achieved low levels of stuttering; maintained for 3 participants 12 months post treatment. - Program took longer to deliver via telehealth - Parents of 3 participants satisfied |
| Lewis et al. [20] | Child stutterers (*n*=10 controls; 8 treatment) | Efficacy of telehealth delivery of established early stuttering intervention versus no treatment. | RCT | Telephone  Audiotape  Video recorded demonstrations | - Telehealth group showed 73% reduction in stuttering (*p*=.02). - 6 children achieved 80% reduction at 9 months post - Parents were satisfied with the telehealth delivery and outcomes |
| O’Brian, et al. [21] | Adult stutterers (*n*=10) | Trial telehealth delivery of established stuttering intervention | Case studies | Audio recordings  Telephone | - Stuttering reduction for group 82% post-intervention, 74% 6 months post. - All participants increased speech rate |
| Carey et al. [25] | Adult stutterers (*n*=20 f-f; 20 telehealth treatment) | Comparative efficacy of telehealth versus f-f delivery of established stuttering intervention | Non-inferiority RCT | Telephone  Voice mail to record speech samples | - NS difference in stuttering frequency immediately, 6, 9 and 12 months post-treatment and at months. - Significantly less contact time required for telehealth - NS difference in speech naturalness, treatment satisfaction and self-reported stuttering severity. |
| Carey et al. [22] | Adolescent stutterers (*n* = 3) | Evaluate telehealth adaptation of adult stuttering intervention for adolescents | One condition repeated measures | Computer with internet access  Webcam, Skype™ at participant site | - Reduction in percent syllables stuttered immediately post-treatment, 6 and 12 months post entry to maintenance - Improvements in self rated severity of stuttering: typical and worst - Achieved speech naturalness within the range of matched controls - Reductions in situation avoidance - Positive feedback about webcam delivery from parents and participants, technical difficulties a disadvantage |
| Carey et al. [23] | Adolescent stutterers (*n* = 14) | Trial delivery of program with evidence of effectiveness for adults to adolescents via telehealth | One condition repeated measures | Computer with internet access  Webcam | - Significant reduction in percent syllables stuttered immediately post-treatment and 12 months post- entry to maintenance - Reduction in self rated severity of stuttering: typical and worst - Increases in speech satisfaction ratings - No difference in speech naturalness with matched controls - Reduction in anxiety for 1 of two participants with problem anxiety scores - Significant reduction in self-reported impact of stuttering - Positive feedback about webcam delivery from parents and participants |
| O’Brian et al. [21] | Child stutterers (*n*=3) | Trial telehealth delivery of established early stuttering intervention | Case studies | Computer with internet access  Webcam | - Reductions to <2% post-treatment; 1% or less 6 months post - Webcam practical and viable, and acceptable to parents |
| Burns et al. [42] | Adults with head & neck cancer  (*n* = 18) | Feasibility of remote clinical management | Descriptive | Videoconference units (both sites)  Hand held medical camera | - Successful clinical management at the remote location - Clinicians reported ease of equipment use - Patients reported comfort with delivery mode, and willingness to use it in the future |
| Ward et al. [35] | Adults post laryngectomy  (*n* = 10) | Comparative feasibility of communication and swallowing assessment through telerehabilitation vs f-f | Comparative study with concurrent control | Portable telerehabilitation units – PC-based VC system  via 3G phone network  Free standing camera with high video resolution | - Clinically acceptable agreement (>80%) for most swallowing and communication variables - Clinically acceptable agreement not reached for some variables requiring visualisation of the oral cavity, stoma or voice prosthesis - Image quality rated as sufficient for clinical decision making by remote clinician |
| Ward et al. [36] | Adults post laryngectomy  (*n* = 20) | Comparative validity of communication and swallowing assessment through telerehabilitation vs f-f | 2 condition repeated measures | Telerehabilitation units – PC-based VC system with audio and video recording  Web cameras  Headphone / microphone headsets | - Clinically acceptable agreement (>80%) for oro-motor function, swallowing status and communication ability - Remote clinician experienced difficulty visualising the stoma - High patient satisfaction - Lower (70%) clinician satisfaction |
| Constantinescu et al. [38] | Adult with Parkinson’s Disease (*n* = 1) | Investigate the validity and feasibility of voice therapy delivered through telerehabilitation | Case study | Telerehabilitation unit – PC-based VC system; parallel systems set up in clinic and patient’s home | - Substantial gains on perceptual measures of voice and speech, except for precision of articulation - Participant reported high satisfaction |
| Constantinescu et al. [29] | Adults with Parkinson’s Disease  (*n* = 61) | Comparative validity and reliability of speech and voice assessment through telerehabilitation vs f-f | RCT | Telerehabilitation unit – PC-based VC system with store & forward | - Good agreement for assessment of voice, oromotor parameters, articulatory precision, & speech intelligibility (80% close agreement) - Poor agreement for 7 voice parameters |
| Constantinescu et al. [30] | Adults with Parkinson’s Disease  (*n* = 34) | Comparative validity and reliability of established treatment for speech and voice disorder | Non-inferiority RCT | Telerehabilitation unit – PC-based VC system with real time, and store & forward | - NS difference between conditions for primary outcome measure (mean change in sound pressure level during a monologue) - High participant satisfaction |
| Theodoros et al. [34] | Adults with Parkinson’s Disease  (*n* = 10) | Trial telehealth delivery of established treatment for speech and voice disorder | Case studies | Telerehabilitation unit – PC-based VC system with real time, and store & forward  Speech processor | - Improvement on pre and post measures of sound pressure level (p < 0.01) and pitch range (p < 0.05) - High participant satisfaction (70%) |
| Sharma et al. [33] | Simulated adults with dysphagia  (*n* = 10) | Comparative feasibility and validity of dysphagia assessment through telerehabilitation vs f-f | RCT | Telerehabilitation unit – PC-based VC system over wireless connection with real time, and store & forward  Fixed and free standing video cameras  Finger pulse oximeter | - High – excellent agreement on all parameters of the clinical swallowing assessment |
| Ward et al. [37] | Adults with dysphagia (*n* = 40) | Comparative validity and reliability of speech assessment through telerehabilitation vs f-f | 2 condition repeated measures | Telerehabilitation unit – PC-based VC system over wireless connection with real time, and store & forward | - High - excellent acceptable levels of agreement across all parameters of the clinical swallowing assessment |
| Hill et al. [32] | Adolescents/ adults with acquired speech apraxia  (*n* = 11) | Comparative validity and reliability of speech assessment through telerehabilitation vs f-f | Non-inferiority RCT | Telerehabilitation unit – PC-based VC system with real time, and store & forward | - NS difference between conditions for assessment results - Moderate to good a greement across conditions (weighted kappa) - Descriptive statistics for inter- and intra-rater agreement were adequate |
| Hill et al. [31] | Adults with acquired dysarthria  (n = 19) | Comparative feasibility and effectiveness of motor speech assessment through telerehabilitation vs f-f | RCT Counterbalanced, repeated measures design. | Telerehabilitation unit – PC-based VC system with real time, and store and forward  Web cameras  Headset microphone | - Good agreement on measures of severity of dysarthria, speech intelligibility and most perceptual ratings - Clinically acceptable agreement not reached on some assessment parameters (ratings of nasality, voice volume and oro-motor skills) |
| Theodoras et al. [39] | Adults with acquired aphasia (n = 32) | Compare the validity and reliability of speech and language assessment through telerehabilitation vs f-f | 2 condition repeated measures | Telerehabilitation unit – PC-based VC system with real time, and store and forward  Web cameras  Headset microphone | - Good to high agreement on test scores and agreement across telerehabilitation and f-f clinicians - Participants reported high satisfaction comfort and audio- and visual quality. |
| Hill et al. [40] | Adults with acquired aphasia (n = 32) | Determine if severity of aphasia influences the accuracy of assessment via telehealth | 2 condition repeated measures | Telerehabilitation unit – PC-based VC system with real time, and store and forward  Web cameras  Headset microphone. | - Most language scale outcomes were not influenced by assessment mode across severity levels with the exception of two sub-scales - Scores were comparable within severity levels |
| Waite et al. [28] | Children with speech disorders  (n = 20) | Comparative validity and reliability of speech intelligibility and oro-motor screening through telehealth vs f-f | RCT | Telehealth unit - PC-based VC system with real-time and store and forward  Web cameras  Headphone / microphone headsets | - Good agreement on ratings of speech intelligibility (100% close agreement) - Low levels of agreement for some oro-motor parameters |
| Waite et al.[27] | Children with language disorders  (n = 25) | Comparative validity and reliability of language assessment via telehealth vs f-f | RCT | Telehealth unit – PC-based VC system with real-time and store and forward  Web cameras  Headphone / microphone headsets | - NS difference in total raw scores and scaled scores between conditions - Good agreement for all subtests (Kappa > .90) |
| Waite et al. [26] | Children with speech disorders  (*n* = 6) | Comparative feasibility of assessment of childhood speech disorders through telehealth vs f-f | 2 condition repeated measures | Telerehabilitation unit – PC-based VC system with real time, and store & forward | - Good agreement for single-word articulation (92%) speech intelligibility (100%) and oro-motor tasks (91%) - Good inter- and intra-rater agreement for online ratings on most measures |
| *Physiotherapy* | | | | | |
| Russell et al. [44] | Adults with ankle pain  (*n*=15) | Comparative validity and reliability of physical examination across remote and f-f conditions | 2 condition repeated measures | Telerehabilitation unit – PC-based VC system with motion analysis and recording of assessment | - Good agreement for patho-anatomical diagnoses (93% , χ^2^ = 4.267; *p* < 0.04) - Binary data tests: very strong agreement (χ^2^ = 234.4; *p* < 0.001) - Categorical data tests: very strong agreement (k = 0.92) |
| Russell et al. [47] | Adults with lower limb pain not associated with a (*n*=19) | Comparative validity and reliability of physical examination across remote and f-f conditions | 2 condition repeated measures | Telerehabilitation unit - VC system with motion analysis and recording of assessment | - Exact agreement primary diagnosis 63%+; 79%+ for similar diagnosis - Substantial agreement (kappa 0.61-0.80) for validity - High agreement (kappa 0.81-1.0) for intra- and inter-relater reliability) - High participant satisfaction with remote assessment |
| Russell et al. [41] | Adults who have received total knee arthroplasty  (*n*= 31 telerehabilitation condition; 34 f-f) | Comparative effectiveness of therapy across remote and f-f conditions | Non-inferiority RCT | Telerehabilitation unit – PC-based VC system; store & forward of high quality video | - NS differences for flexion, extension range of motion, muscle strength, limb girth, pain, timed up-and-go test, quality of life, and clinical gait - Significant improvements over time for both groups, but greater for telerehabilitation for functional outcomes and stiffness measures - NS difference in compliance - Telerehabilitation group reported high levels of satisfaction |
| Lade et al. [43] | Adults with elbow injury  (*n* = 10) | Comparative validity and reliability of physical examination across remote and f-f conditions | 2 condition repeated measures | Telerehabilitation unit – PC-based VC system with motion analysis and recording of assessment | - Good agreement for patho-anatomical diagnoses (73%) - Binary data tests: Varied validity (46%-90%); good intra-rater reliability (81% - 98%); moderate-good inter-rater reliability (68% - 98%). - Categorical data tests: moderate-good validity (71% & 86%); good intra-rater (88% & 95%) and inter-rater reliability (85% & 94%). |
| Steele et al. [45] | Adults with shoulder problems  (*n* = 22) | Comparative validity and reliability of diagnosis across remote and f-f conditions | 2 condition repeated measures | Telerehabilitation unit – PC-based VC system with motion analysis and recording of assessment | - Good agreement for patho-anatomical diagnoses (60%); moderate inter-rater (74%) and excellent intra-rater (100%) reliability - Substantial agreement for primary systems diagnosis (78.6%); good for both intra- and inter-rater reliability (82.1%) - Physical examination: poor to good agreement (56.1%- 87.4%), moderate to good reliability (67%-98%) - Fair agreement for pain ratings; substantial for severity; inter- and intra-rater reliability high - High patient satisfaction |
| Russell et al. [46] | Adults with Parkinson’s Disease (*n*=12) | Comparative validity and reliability of physical assessments across telerehabilitation and f-f conditions | 2 condition repeated measures measures) | Telerehabilitation unit – VC system with optical calibrated assessment tools and recording of assessment  3G internet connection | - High level of inter- and intrarater reliability - Good agreement across conditions; weighed kappa scores of 0.90 for all ordinal scale items - Limits of agreement clinically acceptable for functional lateral reach, Timed Stance test, Timed Up and Go test |
| Cox et al. [49] | Adults with Cystic Fibrosis (*n*=10) | Comparison of performance and reliability across remote and f-f supervison of exercise test | 2 condition repeated measures | VC unit comprising desktop with internet access and webcam | - Test completed by all participants across conditions - NS difference across conditions in physiological responses - Good agreement across conditions for measures taken by clinicians - Sound quality rated poorly for remote condition - No differences for client ability to interact with clinician or comfort |
| Holland et al. [50] | Adults with chronic obstructive pulmonary disease (*n*=8) | Feasibility of home telerehabilitation; supervised aerobic training twice weekly for 8 weeks | Descriptive | Exercise bike  Tablet computer with webcam for VC  Pulse oximeter - display visibly on VC | - 76% attendance - Excellent ratings for system usability via university network (94/100), lower usability ratings via hospital network (59/100) - Clinically significant improvements for 5 participants, no adverse events |
| *Occupational Therapy* | | | | | |
| Hoffmann et al. [48] | Adults with Parkinson’s Disease  (*n*=12) | Comparative validity and reliability of hand function assessment across remote and f-f conditions | RCT | Telerehabilitation unit – PC-based VC system with suite of calibrated assessment tools | - Activities of Daily Living: good exact agreement across conditions (75%-100%); high inter-rater agreement between f-f and observer (ICC=.90) & remote and observer (ICC=.94) - Exact agreement on Parkinson’s Disease rating scale variable (42%-100%); f-f interater agreement (ICC=.90); telerehabilitation (ICC=.80) - Hand function measures good agreement, with high inter-rater agreement in both conditions (ICCs>.99) |
| *Podiatry* | | | | | |
| Manuel [51] | Adult with diabetic foor or leg ulcers  (*n*=13) | Demonstrate ulcer healing rates following service combining remote wound consultations & local support | Descriptive | Videoconferencing, email, hi fidelity digital imagery | - Average size of wounds decreased from 81 mm^2^ to 13 mm^2^, with 3 healing. - Average healing rate per week was 18.2%. - Little resistance by the local wound care team |
| *Audiology* | | | | | |
| Pearce et al. [52] | 6 audiology patients | Explore the feasibility of delivering audiology services by tele-audiology | Case studies | Laptop with audiometer & interfaced assessment tools; wireless broadband mobile card, video-conference units | - Successful hearing assessment, hearing aid adjustment, rehabilitation counselling with clinician instructing a remote assistant in the direct assessment |
|  |  |  |  |  |  |
| *Multidisciplinary* |  |  |  |  |  |
| Crotty et al. [53] | Community patients requiring rehabilitation (*n* = 61) & rural nursing home residents with a history of a recent injury, fall or hospitalisation (*n=*43) | Examine the feasibility of telerehabilitation in the home instead of conventional face-to-face rehabilitations | Descriptive | Videoconferencing – using off the shelf technology, tablet devices, desktop video recorder, document camera, activity monitoring app, interactive whiteboard | - Most participants completed intervention (up to 8 weeks), receiving an average of 10.9 video-consultations and 5.9 home visits - Participants felt they had achieved 50% - 75% of goals - Participants were generally comfortable with the technology, with satisfaction associated with greater number of and time in video-conferencing sessions - In 63% of 366 occasions of service, clinicians were equally satisfied with video-conferencing and face-to-face sessions - Clinicians found video-conferencing better than telephone consultations and equivalent to face-to-face - Reductions in travel time were estimated, which were greater for speech pathologists than physiotherapists. |

Note: f-f = face-to-face; RCT = Randomised Controlled Trial; PC = personal computer; VC = videoconferencing; ICC = interclass correlations; NS = nonsignificant

Table 2.

*Summaries of studies primarily of attitudes and opinions about ehealth*

| *Study* | *Participant Group* | *Focus* | *Design* | *Technology* | *Key Outcomes* |  |
| --- | --- | --- | --- | --- | --- | --- |
| *Occupational Therapy* | | | | | |  |
| Taylor & Lee [57] | Therapists in Western Australia (*n*=413, 82 rural) | Access to and usage of ICT.  Influence of availability and support on recruitment and retention in rural areas. | Mailed written questionnaire. | Computers, email, internet, teleconferencing and videoconferencing. | - Greater use of ICT by rural therapists; predominant usage for work management tasks and professional development. - Videoconferencing for service delivery by 51.5% of rural therapists and 34.2% of non-rural therapists (NS). - 45.5% reported access to ICT influenced their decision to stay in a rural job. |  |
| Hoffmann & Cantoni [56] | Therapists in Queensland (*n*=39), providing services to clients with neurological conditions. | Availability of services to clients with neurological conditions living in Queensland (outside Brisbane). Perspectives on barriers to service delivery and current usage of ICT. | E-mailed written questionnaire. | Telephone, fax, email, internet and videoconferencing. | - Most frequently saw clients in their homes for varied services (home modifications, equipment, education, ADL assessments, retraining). - Frequent barriers to service provision were travel distance, large workloads and limited resources. - The technologies surveyed were available to most, but few used them for other than professional development; reliance on phone for service delivery activities. |  |
| Chedid et al. [55] | Rural NSW therapists (*n*=13), working with people with disability. | Impact of ICT on the workplace practices of rural occupational therapists. | Qualitative grounded theory; data collected from telephone interviews. | Computer, laptop, internet, email, telephone, mobile phone, videoconferencing, tablets and digital camera. | - Videoconferencing accessible to most therapists (*n*=10); most use for professional development. - A few therapists used tablets, but not supported by workplace. - Therapist barriers were age (<30 years more likely to use), knowledge and preferences. - Workplace barriers were lack of ICT support and training, access to ICT when travelling. - Community barriers were lack of or costly ICT access, and perceptions of client acceptance, including an expectation that clinicians will incorporate their clients’ ICT use in their work. |  |
|  |  |  |  |  |  | |
|  |  |  |  |  |  | |
| *Speech pathology* | | | | | | |
| Dunkley et al. [54] | Rural NSW residents (*n* = 43, 41 female); aged 35–44yrs.  Rural NSW therapists (*n* = 49, 47 female); aged 25-29 years; professional experience .5 – 20 years | Access to and attitudes towards ICT in service delivery. | Surveys and follow-up interviews (*n* = 10 residents, 4 speech pathologists) | Web camera, digital versatile (or video) disc (DVD), video camera, videoconferencing, satellite phone, teleconferencing, video cassette recorder (VCR), CD ROM, internet, computer, and email. | - Rural residents reported ready access to computers, DROMs, internet, e-mail, and VCRs or DVDs ; therapists reported access to computers and email only. - Both groups rarely used videoconferencing. - Therapists were very confident and more so than residents in using e-mail, internet, VCRs, DVDs, and video cameras - Rural residents reported more positive attitudes to the use of ICT than perceived by speech pathologists and their own attitudes. - Both groups identified cost as a barrier to uptake of ICT for services and believed it should not be used in place of face-to-face services. | |
| Constantinescu [61] | Rural Queensland parents of children with hearing loss from rural (*n*=13); children aged 6 months -6.5years; therapists delivering Auditory Verbal Therapy via telemedicine (*n*=5). | Satisfaction with telemedicine delivery of therapy 6 months after completion. | Written questionnaires | PC-based videoconferencing (Skype) | - Parents rated audio quality (61%) and video quality (58%) as good or excellent. - Parents identified technical difficulties during sessions (61%), but reported improved comfort with use of the equipment over time. - Parents reported satisfaction with their personal level of comfort and the child’s interaction with the therapist. - Therapists rated audio quality as good (60%) and video quality as good or excellent (80%). - Therapists were comfortable with the telemedicine session and reported rarely experiencing technical problems (80%). | |
| *Podiatry* | | | | | | |
| Barrett et al. [59] | 12 health sites in rural Western Australia: GP practices, podiatry, hospitals, aged care, nursing service, health clinics | Barriers to implementing a telehealth program in a rural location. | Descriptive | Computer with specific software installed and access to the internet | - Barriers were delays installation of software, disease burden of patient group, and workforce shortages. | |
| *Dietetics* | | | | | | |
| O’Sullivan [62] | Western Australia clinicians (*n*=12) | Evaluate use of an on-line electronic record prototype incorporating the Nutritional Care Process and International Dietetics and Nutrition Terminology. | Pre- and post online questionnaires *(n*=12) and focus group (*n*=7). | Electronic record prototype, computer, internet. | - Part implementation reported by 42%. Others reported an intention to implement in the future. - Increase of 17% in confidence with the terminology following the trial. - Respondents (67%) felt that the tool would be more likely to be adopted if available and accessible in the workplace. - Focus group noted advantages of e-health over paper-based records. | |
| *Audiology* | | | | | | |
| Eikelboom & Atlas [58] | 116 Perth audiology clinic patients | Willingness to use and attitudes towards telemedicine | Paper survey administered in clinics | Telemedicine or telehealth (not defined) | - 45% had used the internet for health matters, 25% were aware of telemedicine - Main benefit was reduced waiting time for appointments, 42% willing/ sometimes willing to use telehealth; 30% not willing - Main barrier was preference for f-f. | |
| Meyer et al. [60] | Adults with suspected hearing impairment (*n*=112, 81 males); aged 24-93 yrs. | Actions of individuals who failed a telephone-based hearing screening. | Telephone interview conducted 4-5 months post-screening. | Telescreen; a telephone-based test of hearing. Responses are recorded via a telephone key pad. | - Following a failed telephone-based hearing screen only 36% sought advice from a professional, with approximately half of these receiving a hearing aid. - Individuals were more likely to take action if they had considered hearing aids before the screening, and /or recalled the screening results. | |
| *Multidisciplinary* | | | | | | |
| Usher [63] | 5 Australian Health Professions; general practice, social work, dietetics, physiotherapy and optometry (*n*=746, 59.8% located in major cities). | Usage of web-based health information. | On-line survey | Internet, public health websites. | - Social work and dietetics most frequently recommended health websites to patients (11-20% in a 12 month period) - Health professionals reported that patients rarely brought health information sourced from the internet to consultations; less than 1% across the professions, figures higher for general practice, optometry and physiotherapy. - Internet recommendations from health professionals, internet requests from patients and internet consultations occurred infrequently; results do not reflect international patterns of usage. | |

Note: ADL = activities of daily living; NS = nonsignificant; numbers in brackets correspond to reference in the article.
